# A longitudinal microstructural MRI dataset in healthy C57Bl/6 mice at 9.4 Tesla

**DOI:** 10.1038/s41597-023-01942-5

**Published:** 2023-02-14

**Authors:** Naila Rahman, Kathy Xu, Matthew D. Budde, Arthur Brown, Corey A. Baron

**Affiliations:** 1 Centre for Functional and Metabolic Mapping (CFMM), Robarts Research Institute, University of Western Ontario, London, Ontario Canada; 2 Department of Medical Biophysics, Schulich School of Medicine and Dentistry, University of Western Ontario, London, Ontario Canada; 3 Translational Neuroscience Group, Robarts Research Institute, Schulich School of Medicine and Dentistry, University of Western Ontario, London, Ontario Canada; 4 Department of Neurosurgery, Medical College of Wisconsin, Milwaukee, Wisconsin United States of America; 5 Department of Anatomy and Cell Biology, University of Western Ontario, London, Ontario Canada

**Keywords:** Computational neuroscience, Biomarkers

## Abstract

Multimodal microstructural MRI has shown increased sensitivity and specificity to changes in various brain disease and injury models in the preclinical setting. Here, we present an *in vivo* longitudinal dataset, including a subset of *ex vivo* data, acquired as control data and to investigate microstructural changes in the healthy mouse brain. The dataset consists of structural T2-weighted imaging, magnetization transfer ratio and saturation imaging, and advanced quantitative diffusion MRI (dMRI) methods. The dMRI methods include oscillating gradient spin echo (OGSE) dMRI and microscopic anisotropy (μA) dMRI, which provide additional insight by increasing sensitivity to smaller spatial scales and disentangling fiber orientation dispersion from true microstructural changes, respectively. The technical skills required to analyze microstructural MRI data are complex and include MRI sequence development, acquisition, and computational neuroimaging expertise. Here, we share unprocessed and preprocessed data, and scalar maps of quantitative MRI metrics. We envision utility of this dataset in the microstructural MRI field to develop and test biophysical models, methods that model temporal brain dynamics, and registration and preprocessing pipelines.

## Background & Summary

**Fig. 1:**
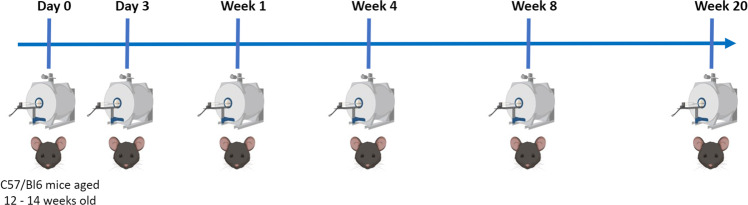
Overview of study design. At Day 0, all mice were 12–14 weeks old. Each C57Bl/6 mouse (n = 12, six males and six females) was scanned at 6 different timepoints, comprising a total of 72 MRI sessions. After Week 20, four of the mice (two males and two females) underwent *ex vivo* imaging.

Multimodal microstructural MRI has shown increased sensitivity and specificity to microstructural changes in various brain disease and injury models in the preclinical setting. Here, we present an *in vivo* longitudinal imaging dataset in the healthy mouse brain, which includes structural T2-weighted, magnetization transfer (MT), and advanced diffusion MRI (dMRI) data. There were no hardware or software changes during data acquisition, and all protocols for a single timepoint in each mouse were acquired in the same session. Each of 12 C57Bl/6 mice were scanned at 6 different timepoints, between 3–8 months of age (Fig. 1). Importantly, this dataset provides imaging data in the same mice over time, which provides greater statistical power compared to cross-sectional studies, to detect changes in brain maturation, as myelination continues to increase between three and six months^1^. The data were acquired with the goals of forming a control dataset and investigating microstructural changes in the healthy mouse brain.

To optimize potential applications of this dataset, we provide dMRI pulse sequences and protocols, source data (DICOM format), code to process source data, unprocessed and preprocessed data (NIFTI format), and quantitative MRI metric maps. We have made this dataset publicly available as other groups may not have access to all resources required to undertake a longitudinal MRI study. This includes hardware, software (specifically custom pulse sequences to implement advanced dMRI protocols), and time/personnel required. Analysis of test-retest reproducibility of the MRI metrics, using a subset of the data, have been published elsewhere^2,3^. We envision utility of this dataset in the microstructural MRI field to develop and test methods that model temporal brain dynamics, registration and preprocessing pipelines, and biophysical models of brain microstructure. Sex and age-dependent differences can be investigated, as the dataset includes an equal number of male and female age-matched mice. *In vivo* to *ex vivo* changes, arising from perfusion and fixation processes, can be explored, as a subset of *ex vivo* data has been included.

MT imaging has been used extensively to investigate changes in myelin content and integrity^4,5^. The MT imaging protocol applied here enables computation of the widely used MT ratio (MTR), and the more recently developed MT saturation index (MTsat)^6^. As MTR is confounded by T1 effects, flip angle inhomogeneities, and choice of sequence parameters, MTsat was developed to reduce T1 dependence and improve specificity to myelin, while maintaining a feasible scan time. MTsat shows higher white matter contrast in the brain than MTR^3,6^, and has been shown to correlate more with disability metrics than MTR in patients with multiple sclerosis^7^.

Developing advanced dMRI techniques, beyond the conventional diffusion tensor imaging (DTI) model, is currently of broad interest, as DTI lacks the specificity to identify unique microstructural environments^8^. The advanced dMRI methods applied here include oscillating gradient spin echo (OGSE) dMRI^9,10^, implemented by varying the oscillating gradient frequency, and microscopic anisotropy (μA) dMRI^11–14^, implemented via tensor valued diffusion encoding. In addition to advanced dMRI metrics, traditional DTI metrics are also provided. OGSE dMRI provides additional insight, compared to conventional dMRI, by increasing sensitivity to smaller spatial scales. This is a robust dataset to explore the frequency dependence of OGSE dMRI metrics, which may provide insight into the relevant mesoscopic structures affecting water diffusion^15^. Evidence of a linear dependence of mean diffusivity on the square root of OGSE frequency has been demonstrated in healthy and globally ischaemic rodent brain tissue^16^ and in healthy human white matter^17^. In contrast to the widely used fractional anisotropy metric (FA)^8^, which confounds true microstructural changes with fiber orientation dispersion, the microscopic anisotropy (μA) metric quantifies water diffusion anisotropy independent of orientation dispersion^11,18,19^. Importantly, μA dMRI can provide estimates of cell shape^11,18,20–25^. Additionally, diffusional kurtosis estimated from the μA protocol includes linear kurtosis (arising from the linear tensor encoding (LTE) acquisitions) and isotropic kurtosis (arising from the spherical tensor encoding (STE) acquisitions), which can be related to cell size heterogeneity^20^.

As myelin is MR-invisible in diffusion-weighted scans, recent studies have applied both dMRI and MT methods for a more comprehensive view of microstructural changes^26–28^. Thus, there may be interest in investigating longitudinal changes by jointly assessing MT and dMRI data, and additionally testing biophysical models using the combined OGSE, µA, and MT data.

## Methods

### Subjects

All animal procedures were approved by the University of Western Ontario Animal Care Committee and were consistent with guidelines established by the Canadian Council on Animal Care. Twelve adult C57Bl/6 mice (six male and six female) were scanned at six timepoints. They were between 12–14 weeks old at the first timepoint (Fig. 1). Before scanning, anesthesia was induced by placing the animals in an induction chamber with 4% isoflurane and an oxygen flow rate of 1.5 L/min. Following induction, isoflurane was maintained during the imaging session at 1.8% with an oxygen flow rate of 1.5 L/min through a custom-built nose cone. At the end of the study, the mice were euthanized. The mice were anesthetized with ketamine/xylazine (2:1) and then underwent trans-cardiac perfusion with ice-cold saline, followed by 4% paraformaldehyde in phosphate-buffer saline (PBS).

### *In vivo* MRI Acquisition

**Fig. 2:**
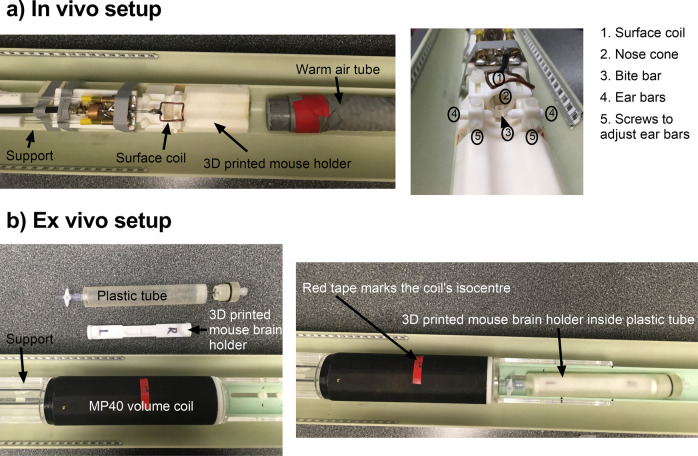
Schematic of experimental setup for *in vivo* and *ex vivo* imaging sessions. (**a**) *In vivo* setup showing the 3D printed mouse holder and surface coil securely attached to a support. The cross-section of the mouse holder depicts how the mouse is secured in place with a nose cone, bite bar, and ear bars. (**b**) *Ex vivo* setup showing the 3D printed mouse brain holder, which can hold two extracted brains, and the 3D printed plastic container, which holds the mouse holder and is filled with Christo-lube. Both the mouse brain holder and container were custom designed to fit in the MP30 volume coil. The MP30 volume coil is securely attached to the support, with the isocentre marked in red.

*In vivo* MRI experiments were performed on a 9.4 Tesla (T) Bruker small animal scanner, running ParaVision 6.0.1, equipped with a gradient coil set of 1 T/m strength (slew rate = 4100 T/m/s). A single channel transceive surface coil (20 mm × 25 mm), built in-house, was fixed in place directly above the mouse head to maximize signal-to-noise ratio (SNR). The mouse holder (which included ear bars and a bite bar), nose cone, and surface coil were fixed onto a support, which was placed into the scanner (Fig. 2a). This ensured consistent positioning of the mouse head in the scanner at each session. 30 slices, with a slice thickness of 400 µm (anatomical scans) or 500 µm (diffusion-weighted scans), were required for full brain acquisition for all protocols. Anatomical images were acquired at each session for each subject using a T2-weighted TurboRARE sequence. A brief overview of the protocols is given in Table 1.

**Table 1:** Brief sequence details for *in vivo* imaging. For a full list of parameters, the exported protocols are included in the repository.

Sequence	FoV (mm^3^)	Slice Thickness (µm)	In-plane Resolution (µm^2^)	TE/TR (ms)	α (°)	Averages	Scan Time (min.)	Notes	
T2 weighted TurboRARE	19.2 × 14.4 × 15	500	150 × 150	40/5000	90	16	22		
MT Imaging FLASH-3D	19.2 × 14.4 × 12	400	150 × 150	2.75/25	9	12	18	MT pulse on (MTw)	
	19.2 × 14.4 × 12	400	150 × 150	2.75/25	9	12	18	Reference PD-weighted scan (PDw)	
								MT pulse off	
	19.2 × 14.4 × 12	400	150 × 150	2.75/8.5	20	12	6	Reference T1-weighted scan (T1w)	
								MT pulse off	
	19.2 × 14.4 × 12	400	300 × 300	3/(TR_1_ = 20, TR_2 _ = 100)	60	1	1	B1 map	
OGSE dMRI Single-shot EPI	19.2 × 14.4 × 15	500	175 × 200	39.2/10000	90	5	45	b-value (s/mm^2^)	# of directions
								0	10
								800	10
µA dMRI Single-shot EPI	19.2 × 14.4 × 15	500	175 × 200	26.8/10000	90	3	45	b-value (s/mm^2^)	# of directions
								0	8
								1000	12 LTE + 12 STE
								2000	30 LTE + 30 STE

#### OGSE and µA dMRI

Each dMRI protocol was acquired with single-shot spin echo echo-planar-imaging (EPI) readout with partial Fourier imaging in the phase encode direction with 80% of k-space being sampled. For each dMRI protocol, a single reverse phase encoded b = 0 s/mm^2^ volume was acquired at the end of the diffusion sequence for subsequent use in TOPUP^29^ and EDDY^30^, from FMRIB Software Library (FSL, Oxford, UK)^31^, to correct for susceptibility and eddy current induced distortions.

The OGSE dMRI protocol included a PGSE sequence (with gradient duration = 11 ms and diffusion time = 13.8 ms) and four OGSE sequences with oscillating gradient frequencies of 50 Hz, 100 Hz, 145 Hz, and 190 Hz at b = 800 s/mm^2^ (10 directions for each frequency). The lowest OGSE frequency (50 Hz) uses the newly introduced frequency tuned bipolar (FTB) waveforms to reduce TE of the acquisition^32^. The μA sequence was implemented with linear (LTE) and spherical tensor (STE) encodings, as shown in Table 1, at b = 2000 s/mm^2^ (30 directions for each of LTE and STE) and b = 1000 s/mm^2^ (12 directions). Details about gradient waveforms and gradient modulation power spectra for the OGSE and µA protocols implemented here are presented in Rahman *et al*.^2^.

#### MT Imaging

The MT protocol required 50 minutes total scan time and comprised three FLASH-3D (fast low angle shot) scans and one RF transmit field (B1) map scan acquired using the actual flip-angle imaging (AFI) method^33^ to correct for local variations in flip angle. An MT-weighted scan, and reference T1-weighted and PD-weighted scans (MTw, T1w, and PDw respectively) were acquired by appropriate choice of the repetition time (TR) and the flip angle (α). MT-weighting was achieved by applying an off-resonance Gaussian-shaped RF pulse (12 ms duration, 385° nominal flip angle, 3.5 kHz frequency offset from water resonance, 5 µT RF peak amplitude) prior to the excitation.

### *Ex vivo* MRI Acquisition

*Ex vivo* MRI experiments were performed on a subset of four mice (two male and two female) after the last *in vivo* scan. The mouse IDs of *ex vivo* data are: NR1_F (female), NR2_F (female), NR7_M (male), and NR8_M (male). NR1_F and NR2_F were scanned with the skull attached to the brain to minimize chances of tissue deformation, while NR7_M and NR8_M were scanned with the skull removed.

*Ex vivo* imaging was also performed on the 9.4 Tesla (T) Bruker small animal scanner, running ParaVision 6.0.1, equipped with a gradient coil set of 1 T/m strength (slew rate = 4100 T/m/s). A 3D printed mouse brain holder, holding two mouse brains at a time, was placed into a 3D printed plastic container and submerged with lubricant (Christo-lube MCG 1009; Engineered Custom Lubricants) to avoid magnetic susceptibility-related distortion artifacts (Fig. 2b). The mouse brain holder and container were custom designed to fit in the MP30 volume coil (Agilent, Palo Alto, CA, USA). The container was then slid into the volume coil (fixed on a support) and taped onto the support. The design of the mouse brain holder and container ensured that the mouse brain was positioned at the isocentre of the volume coil and the design of the support ensured consistent positioning of the mouse brain in the scanner at each session. 30 slices, with a slice thickness of 400 µm (anatomical scans) or 500 µm (diffusion-weighted scans), were required for full brain acquisition for all protocols. Anatomical images were acquired for each brain using a T2-weighted TurboRARE sequence. Due to field-of-view (FOV) constraints, one brain was imaged at a single session (although the mouse holder was designed to hold two brains). A brief overview of the protocols is given in Table 2. The total *ex vivo* scan time for each brain was 15 hours.

**Table 2:** Brief sequence details for *ex vivo* imaging. For a full list of parameters, the exported protocols are included in the repository.

Sequence	FoV (mm^3^)	Slice Thick-ness (µm)	In-plane Resolution (µm^2^)	TE/TR (ms)	α (°)	Averages	Scan Time	Notes	
T2 weighted TurboRARE	19.2 × 14.4 × 17.5	500	100 × 100	30/5000	90	48	1h12min		
MT Imaging FLASH-3D	19.2 × 14.4 × 12	400	100 × 100	3.06/30	9	36	1h42min	MT pulse on (MTw)	
	19.2 × 14.4 × 12	400	100 × 100	3.06/30	9	36	1h42min	Reference PD-weighted scan (PDw)	
								MT pulse off	
	19.2 × 14.4 × 12	400	100 × 100	3.06/12	20	36	40 min	Reference T1-weighted scan (T1w)	
								MT pulse off	
	19.2 × 14.4 × 12	400	300 × 300	3/(TR_1_ = 20, TR_2_ = 100)	60	1	1 min	B1 map	
OGSE dMRI Multishot EPI (2 segments)	19.5 × 15 × 15	500	130 × 150	36.4/15000	90	14	6h25min	b-value (s/mm^2^)	# of directions
								0	10
								1600	10
µA dMRI Multishot EPI (2 segments)	19.5 × 15 × 15	500	130 × 150	28.9/10000	90	10	3h34min	b-value (s/mm^2^)	# of directions
								0	4
								1320	6 LTE + 6 STE
								2640	9 LTE + 9 STE
								4000	15 LTE + 15 STE

#### OGSE and µA dMRI

Each dMRI protocol was acquired with multi-shot spin echo echo-planar-imaging (EPI) readout with 2 shots and partial Fourier imaging in the phase encode direction with 75% of k-space being sampled. Reverse phase-encoded volumes were not acquired for *ex vivo* data.

The OGSE dMRI protocol included a PGSE sequence (with gradient duration = 11 ms and diffusion time = 13.8 ms) and four OGSE sequences with oscillating gradient frequencies of 50 Hz, 80 Hz, 115 Hz, and 150 Hz at b = 1600 s/mm^2^ (10 directions for each frequency), with the lowest OGSE frequency using the FTB waveform. The μA sequence was implemented with linear (LTE) and spherical tensor (STE) encodings, as shown in Table 2, at b = 1320 s/mm^2^ (6 directions for each of LTE and STE), b = 2640 s/mm^2^ (9 directions), and b = 4000 s/mm^2^ (15 directions).

#### MT Imaging

MT-weighting was achieved by applying an off-resonance Gaussian-shaped RF pulse, with the same parameters for *in vivo* imaging, prior to the excitation.

### Data analysis pipeline

**Fig. 3:**
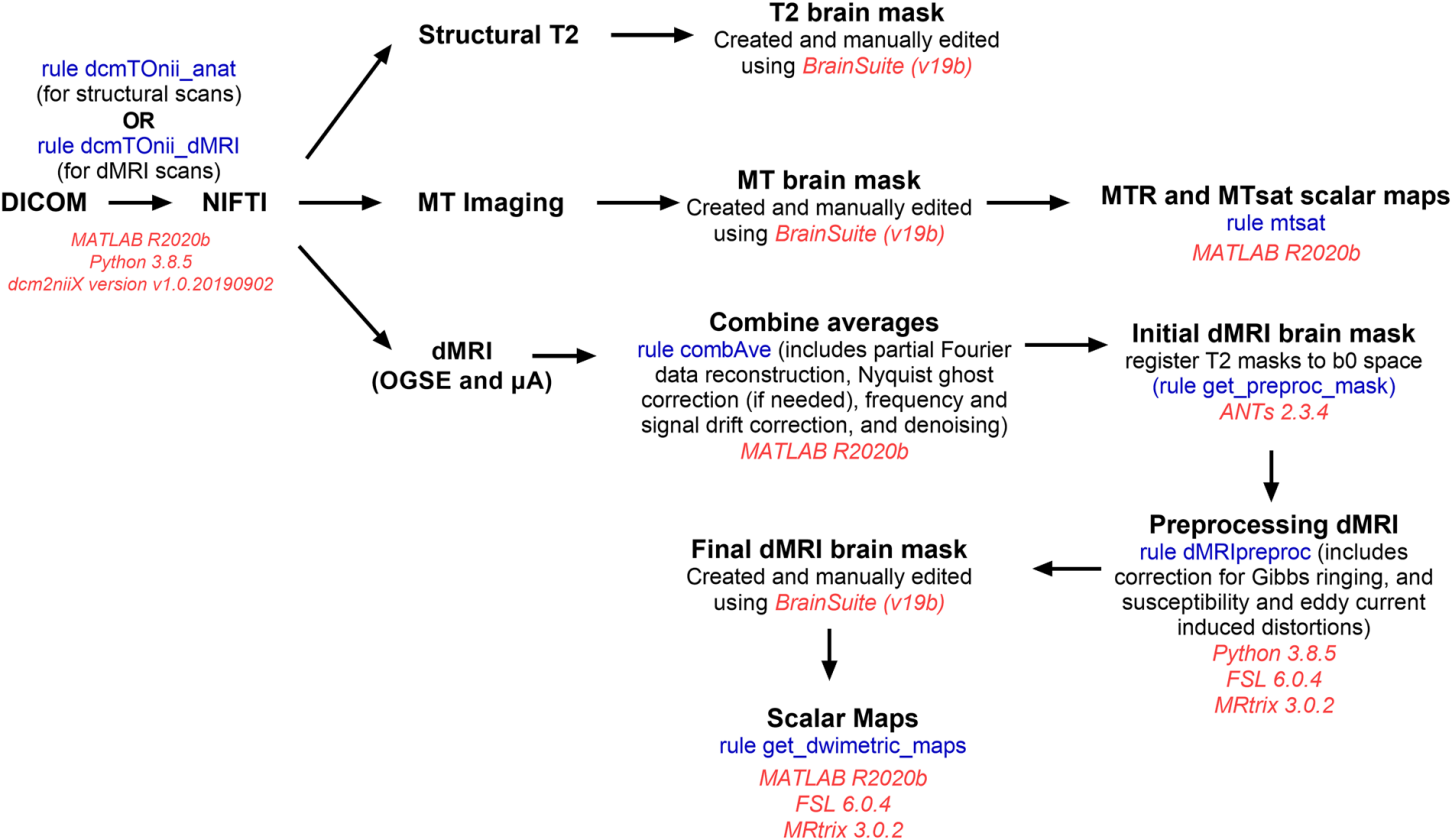
Flowchart outlining data analysis steps from DICOM to scalar map generation. The “rules” listed are those used in the Snakemake pipeline, using Snakemake 3.13.3. The software dependencies and versions of each analysis step are included.

The data analysis pipeline was built using Snakemake^34^ (described in greater detail in the Usage Notes section). A Snakemake workflow defines data analysis in terms of rules that are specified in the “Snakefile.” Fig. 3 outlines the data analysis steps from DICOM to scalar map generation.

#### OGSE and µA dMRI recon and preprocessing

For the dMRI protocols, averages were acquired separately on the scanner and the complex-valued averages were combined using in-house MATLAB code which included reconstruction of partial Fourier data using POCS (Projection onto Convex Sets)^35^, correction for frequency and signal drift associated with gradient coil heating^36^, and Marchenko-Pastur denoising of complex-valued data^37^. If averages were not collected separately, this step can simply be skipped. Importantly, the pipeline can be used for both complex-valued and magnitude data. After the averages were combined, images were preprocessed using Gibbs ringing correction from the MRtrix3 package^38^, followed by TOPUP^29^ and EDDY^30^ from FMRIB Software Library (FSL, Oxford, UK)^31^. Using the data collected with reverse phase-encode blips, the susceptibility-induced off-resonance field was estimated using TOPUP. Then, EDDY was run to correct for eddy current induced distortions (volume-by-volume), perform motion correction, and apply the results from TOPUP.

#### Brain masks

For each protocol, brain masks were produced using the skull stripping tool from BrainSuite (v. 19b)^39^ and manually edited, as needed. For dMRI data, an initial brain mask (after dMRI averages were combined) was created by registering the T2 brain masks to a b = 0 s/mm^2^ volume, using ANTs software^40^. This initial brain mask was required for EDDY in the dMRI preprocessing step. After dMRI preprocessing, a final brain mask was produced and manually edited using BrainSuite. Brain masks for images from the T2, MT, and dMRI protocols have been included in the repository.

#### Scalar map generation

**Fig. 4:**
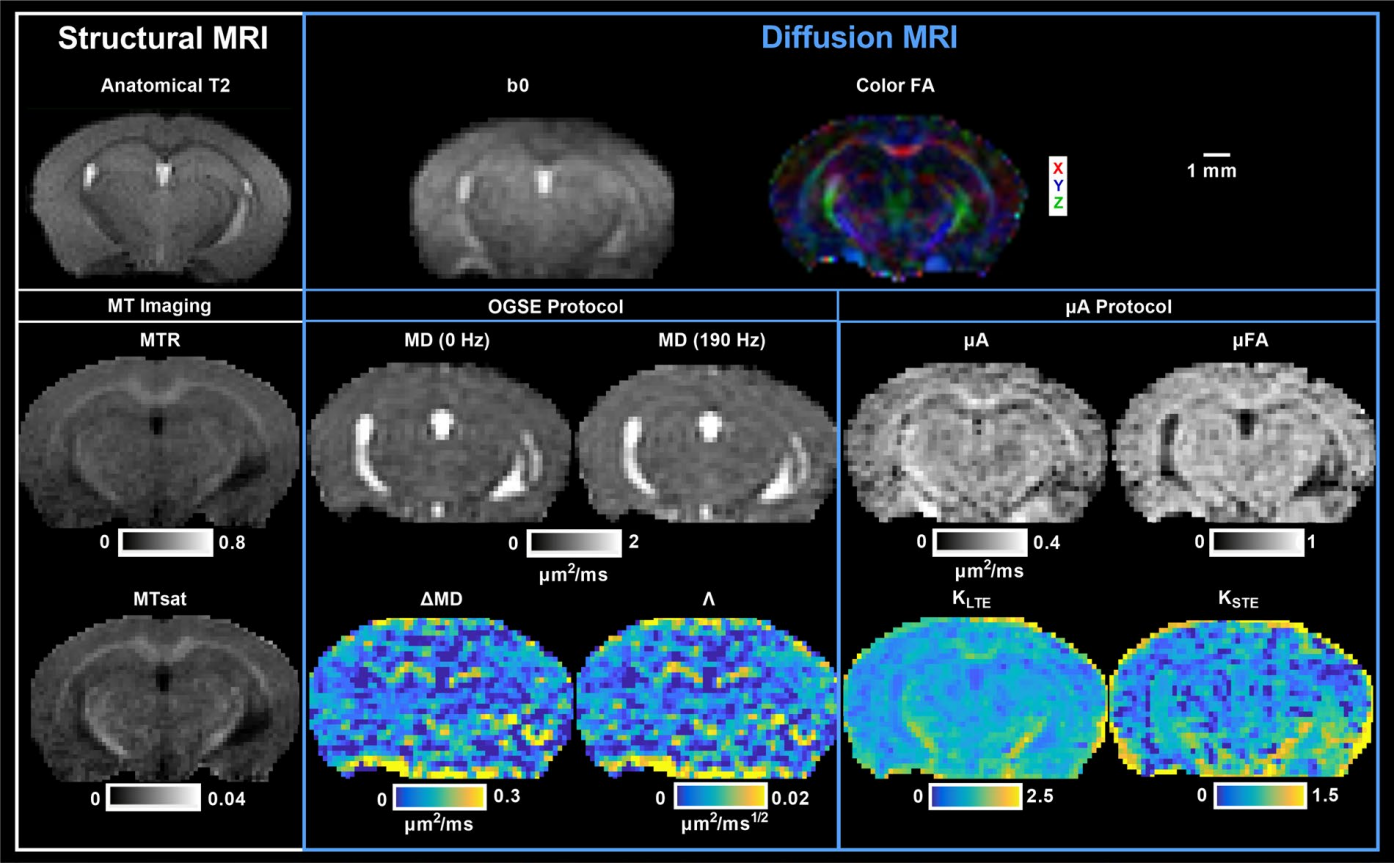
*In vivo* scalar maps. Other DTI metric maps (such as axial and radial diffusivity) are not shown here but have been included in the repository. MTR: magnetization transfer ratio; MTsat: magnetization transfer saturation; FA: fractional anisotropy; MD: mean diffusivity; ΔMD: mean diffusivity difference between MD (190 Hz) and MD (0 Hz); Λ: diffusion dispersion rate; µA: microscopic anisotropy; µFA: microscopic fractional anisotropy; K_LTE_: linear kurtosis calculated from LTE volumes; K_STE_: isotropic kurtosis calculated from STE volumes.

**Fig. 5:**
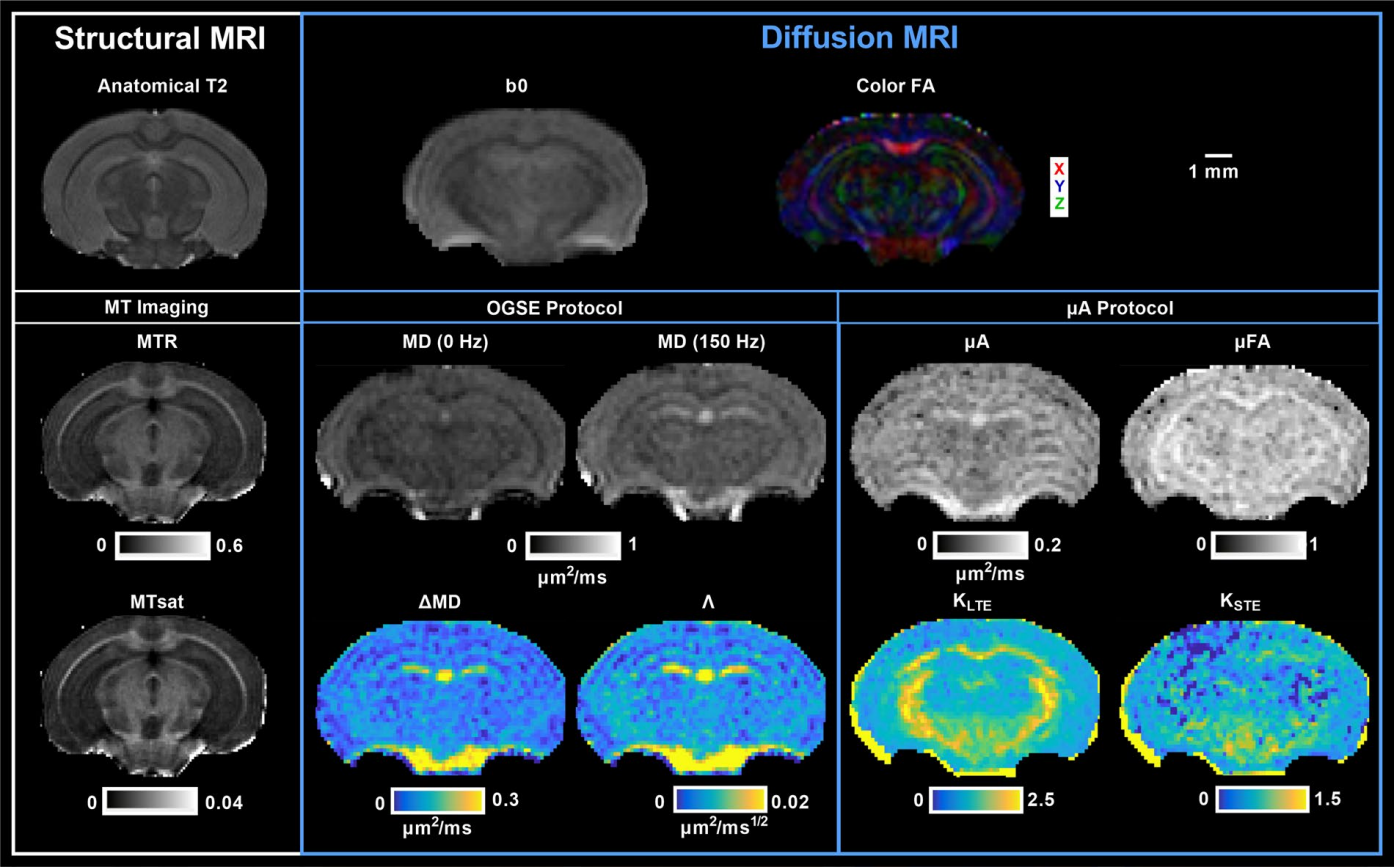
*Ex vivo* scalar maps. Other DTI metric maps (such as axial and radial diffusivity) are not shown here but have been included in the repository. MTR: magnetization transfer ratio; MTsat: magnetization transfer saturation; FA: fractional anisotropy; MD: mean diffusivity; ΔMD: mean diffusivity difference between MD (190 Hz) and MD (0 Hz); Λ: diffusion dispersion rate; µA: microscopic anisotropy; µFA: microscopic fractional anisotropy; K_LTE_: linear kurtosis calculated from LTE volumes; K_STE_: isotropic kurtosis calculated from STE volumes.

Scalar maps are shown in Fig. 4 (*in vivo*) and Fig. 5 (*ex vivo*). The scalar maps provided in the repository are summarized in Supplementary Table 1. Although briefly described here, more details describing the scalar maps are presented in previous reproducibility studies of the data^2,3^.

Scalar maps of MTR and MTsat were generated from the MT protocol. MTw, PDw, and T1w images were used to calculate MTsat maps, following the original method proposed by Helms *et al*.^6^ and outlined by Hagiwara *et al*.^41^ and Rahman *et al*.^3^. Furthermore, B1 maps are available to correct for small residual higher-order dependencies of the MT saturation on the local RF transmit field to further improve spatial uniformity, as suggested by Weiskopf *et al*.^42^.

From the OGSE data, maps of MD at each frequency were generated using MRtrix3^38^. The mean diffusivity difference, ΔMD, was calculated as the difference between MD acquired at the highest frequency (190 Hz (*in vivo*) or 150 Hz (*ex vivo*)) and MD acquired at the lowest frequency (0 Hz). To characterize the power law relationship between MD and OGSE frequency (f)^15^, the slope of linear regression of MD with f,^0.5^ the diffusion dispersion rate (Λ), was calculated. From the µA data, maps of microscopic anisotropy (µA), microscopic fractional anisotropy (µFA), and diffusion kurtosis arising from LTE and STE acquisitions (K_LTE_ and K_STE_ respectively) were generated by fitting the powder-averaged STE and LTE signals versus b-value to the diffusion kurtosis model^14^. The powder-averaged signal, in diffusion MRI, refers to the average signal intensity over all directions in a specific b-shell^11^. As a reference for the OGSE and µA metrics, DTI metrics have been included in the repository.

## Data Records

The datasets, exported MRI protocols, Snakemake pipeline, and in-house MATLAB code are available in the Federated Research Data Repository (FRDR) at 10.20383/103.0594^43^.

Datasets are arranged in ‘Data’ as *mouseID_sex*/*timepoint*/*MRI_contrast*. The *timepoint* includes *in vivo* timepoints (*Day0*, *Day3*, *Week1*, *Week4*, *Week8*, and *Week20*) and the *ex vivo* timepoint (*ex_vivo*). For dMRI data, preprocessed data and scalar maps are arranged in ‘DiffusionDataPreproc’ with the same structure of *mouseID_sex*/*timepoint*/*MRI_contrast*.

***Structural T2-weighted dataset***

**Table:** 

Folder	Filename	Filename Extensions
Data	**T2-weighted scan:** *mouseID_sex*/*timepoint*/T2_TurboRARE_AX150150500_A16/ T2_TurboRARE_AX150150500_A16 (*in vivo)* *mouseID_sex*/*timepoint*/ T2_TurboRARE_AX100100500_A48/ T2_TurboRARE_AX100100500_A48 (*ex vivo)*	.dcm .nii.gz .json _method.json
Data	**Brain mask:** *mouseID_sex*/*timepoint*/T2_TurboRARE_AX150150500_A16/ T2_TurboRARE_AX150150500_A16_mask *(in vivo)* *mouseID_sex*/*timepoint*/ T2_TurboRARE_AX100100500_A48/ T2_TurboRARE_AX100100500_A48_mask *(ex vivo)*	.nii.gz
Data	**T2-weighted scan with brain mask applied:** *mouseID_sex*/*timepoint*/T2_TurboRARE_AX150150500_A16/ T2_TurboRARE_AX150150500_A16_Wmask *(in vivo)* *mouseID_sex*/*timepoint*/ T2_TurboRARE_AX100100500_A48/ T2_TurboRARE_AX100100500_A48_Wmask *(ex vivo)*	.nii.gz

***MT Imaging dataset***

**Table:** 

Folder	Filename	Filename Extensions
Data	**MTw scan:** *mouseID_sex*/*timepoint*/MTon_GRE_3D_150 × 400_12A_5uT_385FA_3500Hz/ MTon_GRE_3D_150 × 400_12A_5uT_385FA_3500Hz *(in vivo)* *mouseID_sex*/*timepoint*/ MTon_GRE_3D_100 × 400_36A_5uT_385FA_3500Hz/ MTon_GRE_3D_100 × 400_36A_5uT_385FA_3500Hz *(ex vivo)*	.dcm .nii.gz .json _method.json _visu_pars.json
Data	**PDw scan:** *mouseID_sex*/*timepoint*/MToff_PD_GRE_3D_150 × 400_12A/MToff_PD_GRE_3D_150 × 400_12A *(in vivo)* *mouseID_sex*/*timepoint*/ MToff_PD_GRE_3D_100 × 400_36A/ MToff_PD_GRE_3D_100 × 400_36A *(ex vivo)*	.dcm .nii.gz .json _method.json _visu_pars.json
Data	**T1w scan:** *mouseID_sex*/*timepoint*/ MToff_T1_GRE_3D_150 × 400_12A/MToff_T1_GRE_3D_150 × 400_12A *(in vivo)* *mouseID_sex*/*timepoint*/ MToff_T1_GRE_3D_100 × 400_36A/ MToff_T1_GRE_3D_100 × 400_36A *(ex vivo)*	.dcm .nii.gz .json _method.json _visu_pars.json
Data	**B1 map data (2 volumes acquired from 2 TRs):** *mouseID_sex*/*timepoint*/rpAFI_mouse_2/rpAFI_mouse_2 *(in vivo* and *ex vivo)*	.dcm .nii.gz .json _method.json _visu_pars.json
Data	**B1 map (from the scanner):** *mouseID_sex*/*timepoint*/rpAFI_mouse_1/rpAFI_mouse_1 *(in vivo* and *ex vivo)*	.dcm .nii.gz .json _method.json _visu_pars.json
Data	**B1 map (resampled to match MTw scan’s resolution):** *mouseID_sex*/*timepoint*/rpAFI_mouse_1/rpAFI_mouse_1_vol2_RS *(in vivo* and *ex vivo)*	.nii.gz
Data	**Text file detailing which B1 map slices have artifacts (0 for slices with the banding artifact and 1 for slices without artifacts):** *mouseID_sex*/*timepoint*/rpAFI_mouse_1/rpAFI_mouse_1 *(in vivo* and *ex vivo)*	.csv
Data	**Brain mask:** *mouseID_sex*/*timepoint*/MTon_GRE_3D_150 × 400_12A_5uT_385FA_3500Hz/MTon_GRE_3D_150 × 400_12A_5uT_385FA_3500Hz_mask *(in vivo)* *mouseID_sex*/*timepoint*/ MTon_GRE_3D_100 × 400_36A_5uT_385FA_3500Hz/ MTon_GRE_3D_100 × 400_36A_5uT_385FA_3500Hz_mask *(ex vivo)*	.nii.gz
Data	**MTR – scalar map:** *mouseID_sex*/*timepoint*/MTon_GRE_3D_150 × 400_12A_5uT_385FA_3500Hz/MTon_GRE_3D_150 × 400_12A_5uT_385FA_3500Hz_mtr *(in vivo)* *mouseID_sex*/*timepoint*/ MTon_GRE_3D_100 × 400_36A_5uT_385FA_3500Hz/ MTon_GRE_3D_100 × 400_36A_5uT_385FA_3500Hz_mtr *(ex vivo)*	.nii.gz
Data	**MTsat – scalar map:** *mouseID_sex*/*timepoint*/MTon_GRE_3D_150 × 400_12A_5uT_385FA_3500Hz/MTon_GRE_3D_150 × 400_12A_5uT_385FA_3500Hz_mtsat *(in vivo)* *mouseID_sex*/*timepoint*/ MTon_GRE_3D_100 × 400_36A_5uT_385FA_3500Hz/ MTon_GRE_3D_100 × 400_36A_5uT_385FA_3500Hz_mtsat *(ex vivo)*	.nii.gz

***OGSE dMRI dataset***

**Table:** 

Folder	Filename	Filename Extensions
Data	**OGSE dMRI scan (complex-valued data):** *mouseID_sex*/*timepoint*/OGSE_5Shapes_1A_5Rep_TR10/OGSE_5Shapes_1A_5Rep_TR10 *(in vivo)* *mouseID_sex*/*timepoint*/OGSE_res130150500/OGSE_res130150500 *(ex vivo)*	.dcm _real.nii.gz _imaginary.nii.gz _real.json _imaginary.json _method.json _visu_pars.json .bmat .bvec .bval
Data	**OGSE dMRI scan (after averages are combined):** *mouseID_sex*/*timepoint*/OGSE_5Shapes_1A_5Rep_TR10/OGSE_5Shapes_1A_5Rep_TR10_aveComb *(in vivo)* *mouseID_sex*/*timepoint*/OGSE_res130150500/OGSE_res130150500_aveComb *(ex vivo)*	.nii.gz .bmat .bvec .bval
Data	**b0 scan acquired with reverse PE (complex-valued data):** *mouseID_sex*/*timepoint*/OGSE_5Shapes_1A_5Rep_TR10_b0_reversePE/OGSE_5Shapes_1A_5Rep_TR10_b0_reversePE_aveComb *(in vivo)* *(*not acquired for *ex vivo)*	.dcm _real.nii.gz _imaginary.nii.gz _real.json _imaginary.json _method.json _visu_pars.json .bmat .bvec .bval
Data	**b0 scan acquired with reverse PE (after averages are combined):** *mouseID_sex*/*timepoint*/OGSE_5Shapes_1A_5Rep_TR10_b0_reversePE/OGSE_5Shapes_1A_5Rep_TR10_b0_reversePE_aveComb *(in vivo)*	.nii.gz .bmat .bvec .bval
Data	**Mean b0 volume extracted from dataset after averages are combined (with normal PE):** *mouseID_sex*/*timepoint*/OGSE_5Shapes_1A_5Rep_TR10/OGSE_5Shapes_1A_5Rep_TR10_aveComb_mean_b0 *(in vivo)*	.nii.gz
DiffusionDataPreproc	**Preprocessed Dataset:** *mouseID_sex*/*timepoint*/OGSE_5Shapes_1A_5Rep_TR10/OGSE_5Shapes_1A_5Rep_TR10_aveComb_preproc *(in vivo)* *mouseID_sex*/*timepoint*/OGSE_res130150500/OGSE_res130150500_aveComb_preproc *(ex vivo)*	.nii.gz .bvec .bval
DiffusionDataPreproc	**Preprocessed Dataset split into separate frequencies (** ***in vivo*** **):** **PGSE or 0 Hz:** *mouseID_sex*/*timepoint*/OGSE_5Shapes_1A_5Rep_TR10/OGSE_5Shapes_1A_5Rep_TR10_aveComb_preproc_f000 **50 Hz OGSE:** *mouseID_sex*/*timepoint*/OGSE_5Shapes_1A_5Rep_TR10/OGSE_5Shapes_1A_5Rep_TR10_aveComb_preproc_f050 **100 Hz OGSE:** *mouseID_sex*/*timepoint*/OGSE_5Shapes_1A_5Rep_TR10/OGSE_5Shapes_1A_5Rep_TR10_aveComb_preproc_f100 **145 Hz OGSE:** *mouseID_sex*/*timepoint*/OGSE_5Shapes_1A_5Rep_TR10/OGSE_5Shapes_1A_5Rep_TR10_aveComb_preproc_f145 **190 Hz OGSE:** *mouseID_sex*/*timepoint*/OGSE_5Shapes_1A_5Rep_TR10/OGSE_5Shapes_1A_5Rep_TR10_aveComb_preproc_f190 **Preprocessed Dataset split into separate frequencies (** ***ex vivo*** **):** **PGSE or 0 Hz:** *mouseID_sex*/*timepoint*/OGSE_res130150500/OGSE_ res130150500_aveComb_preproc_f000 **50 Hz OGSE:** *mouseID_sex*/*timepoint*/OGSE_ res130150500/OGSE_ res130150500_aveComb_preproc_f050 **80 Hz OGSE:** *mouseID_sex*/*timepoint*/OGSE_ res130150500/OGSE_ res130150500_aveComb_preproc_f080 **115 Hz OGSE:** *mouseID_sex*/*timepoint*/OGSE_ res130150500/OGSE_ res130150500_aveComb_preproc_f115 **150 Hz OGSE:** *mouseID_sex*/*timepoint*/OGSE_ res130150500/OGSE_ res130150500_aveComb_preproc_f150	.nii.gz .bvec .bval
DiffusionDataPreproc	**Scalar Maps generated for each frequency (*in vivo*** **and** ***ex vivo*)** **For example, for PGSE or 0 Hz (** ***in vivo*** **):** **Axial Diffusivity (AD):** *mouseID_sex*/*timepoint*/OGSE_5Shapes_1A_5Rep_TR10/OGSE_5Shapes_1A_5Rep_TR10_aveComb_preproc_f000_AD **Radial Diffusivity (RD):** *mouseID_sex*/*timepoint*/OGSE_5Shapes_1A_5Rep_TR10/OGSE_5Shapes_1A_5Rep_TR10_aveComb_preproc_f000_RD **Mean Diffusivity (MD):** *mouseID_sex*/*timepoint*/OGSE_5Shapes_1A_5Rep_TR10/OGSE_5Shapes_1A_5Rep_TR10_aveComb_preproc_f000_MD **Fractional Anisotropy (FA):** *mouseID_sex*/*timepoint*/OGSE_5Shapes_1A_5Rep_TR10/OGSE_5Shapes_1A_5Rep_TR10_aveComb_preproc_f000_FA **Color Fractional Anisotropy (Color FA):** *mouseID_sex*/*timepoint*/OGSE_5Shapes_1A_5Rep_TR10/OGSE_5Shapes_1A_5Rep_TR10_aveComb_preproc_f000_FAvec **Voxelwise Diffusion Dispersion Rate (Λ):** *mouseID_sex*/*timepoint*/OGSE_5Shapes_1A_5Rep_TR10/OGSE_5Shapes_1A_5Rep_TR10_aveComb_preproc_f000_MD_Gfactor	.nii.gz
DiffusionDataPreproc	**Other Scalar Maps** **Mean Diffusivity Difference (between 190 Hz OGSE and PGSE (0 Hz)):** *mouseID_sex*/*timepoint*/OGSE_5Shapes_1A_5Rep_TR10/OGSE_5Shapes_1A_5Rep_TR10_aveComb_preproc_delMD *(in vivo)* *mouseID_sex*/*timepoint*/OGSE_res130150500/OGSE_res130150500_aveComb_preproc_delMD *(ex vivo)*	.nii.gz

*As the same brain mask is used for both dMRI datasets, the brain mask has been included in the µA dMRI dataset*.

***µA dMRI dataset***

**Table:** 

Folder	Filename	Filename Extensions
Data	**µA dMRI scan (complex-valued data):** *mouseID_sex*/*timepoint*/uFA_2Shapes_1A_3Rep_TR10/uFA_2Shapes_1A_3Rep_TR10 *(in vivo)* *mouseID_sex*/*timepoint*/uFA_res130150500/uFA_res130150500 *(ex vivo)*	.dcm _real.nii.gz _imaginary.nii.gz _real.json _imaginary.json _method.json _visu_pars.json .bmat .bvec .bval
Data	**µA dMRI scan (after averages are combined):** *mouseID_sex*/*timepoint*/uFA_2Shapes_1A_3Rep_TR10/ uFA_2Shapes_1A_3Rep_TR10_aveComb *(in vivo)* *mouseID_sex*/*timepoint*/uFA_res130150500/ uFA_res130150500 *(ex vivo)*	.nii.gz .bmat .bvec .bval
Data	**b0 scan acquired with reverse PE (complex-valued data):***mouseID_sex*/*timepoint*/uFA_2Shapes_1A_3Rep_TR10_b0_reversePE/ uFA_2Shapes_1A_3Rep_TR10_b0_reversePE *(in vivo)* *(*not acquired for *ex vivo)*	.dcm _real.nii.gz _imaginary.nii.gz _real.json _imaginary.json _method.json _visu_pars.json .bmat .bvec .bval
Data	**b0 scan acquired with reverse PE (after averages are combined):** *mouseID_sex*/*timepoint*/uFA_2Shapes_1A_3Rep_TR10_b0_reversePE/ uFA_2Shapes_1A_3Rep_TR10_b0_reversePE_aveComb *(in vivo)*	.nii.gz .bmat .bvec .bval
Data	**Mean b0 volume extracted from dataset after averages are combined (normal PE):** *mouseID_sex*/*timepoint*/uFA_2Shapes_1A_3Rep_TR10/uFA_2Shapes_1A_3Rep_TR10_aveComb_mean_b0 *(in vivo)*	.nii.gz
DiffusionDataPreproc	**Preprocessed Dataset:** *mouseID_sex*/*timepoint*/uFA_2Shapes_1A_3Rep_TR10/uFA_2Shapes_1A_3Rep_TR10_aveComb_preproc *(in vivo)* *mouseID_sex*/*timepoint*/uFA_res130150500/uFA_res130150500_aveComb_preproc *(ex vivo)*	.nii.gz .bvec .bval .isiso
DiffusionDataPreproc	**Scalar maps (** ***in vivo*** **):** (for *ex vivo* scalar maps, replace ‘uFA_2Shapes_1A_3Rep_TR10/uFA_2Shapes_1A_3Rep_TR10’ with ‘uFA_res130150500/uFA_res130150500’) **Axial Diffusivity (AD) – acquired with b1000 LTE volumes:** *mouseID_sex*/*timepoint*/uFA_2Shapes_1A_3Rep_TR10/uFA_2Shapes_1A_3Rep_TR10_aveComb_preproc_AD **Radial Diffusivity (RD) – acquired with b1000 LTE volumes:***mouseID_sex*/*timepoint*/uFA_2Shapes_1A_3Rep_TR10/uFA_2Shapes_1A_3Rep_TR10_aveComb_preproc_RD **Mean Diffusivity (MD) – acquired with b1000 LTE volumes:***mouseID_sex*/*timepoint*/uFA_2Shapes_1A_3Rep_TR10/uFA_2Shapes_1A_3Rep_TR10_aveComb_preproc_MD **Fractional Anisotropy (FA) – acquired with b1000 LTE volumes:***mouseID_sex*/*timepoint*/uFA_2Shapes_1A_3Rep_TR10/uFA_2Shapes_1A_3Rep_TR10_aveComb_preproc_FA **Fractional Anisotropy (FA) – acquired with b2000 LTE volumes:** *mouseID_sex*/*timepoint*/uFA_2Shapes_1A_3Rep_TR10/uFA_2Shapes_1A_3Rep_TR10_aveComb_preproc_b2000_FA**Color Fractional Anisotropy (FA) – acquired with b1000 LTE volumes:** *mouseID_sex*/*timepoint*/uFA_2Shapes_1A_3Rep_TR10/uFA_2Shapes_1A_3Rep_TR10_aveComb_preproc_b1000_FAvec **Color Fractional Anisotropy (FA) – acquired with b2000 LTE volumes:** *mouseID_sex*/*timepoint*/uFA_2Shapes_1A_3Rep_TR10/uFA_2Shapes_1A_3Rep_TR10_aveComb_preproc_b2000_FAvec **Microscopic Anisotropy (µA):** *mouseID_sex*/*timepoint*/uFA_2Shapes_1A_3Rep_TR10/uFA_2Shapes_1A_3Rep_TR10_aveComb_preproc_uA**Microscopic Fractional Anisotropy (µFA):** *mouseID_sex*/*timepoint*/uFA_2Shapes_1A_3Rep_TR10/uFA_2Shapes_1A_3Rep_TR10_aveComb_preproc_uFA **Linear Kurtosis – calculated from LTE volumes (KLTE):** *mouseID_sex*/*timepoint*/uFA_2Shapes_1A_3Rep_TR10/uFA_2Shapes_1A_3Rep_TR10_aveComb_preproc_Klin **Isotropic Kurtosis – calculated from STE volumes (KSTE):** *mouseID_sex*/*timepoint*/uFA_2Shapes_1A_3Rep_TR10/uFA_2Shapes_1A_3Rep_TR10_aveComb_preproc_Kiso	.nii.gz
DiffusionDataPreproc	**Initial brain mask (for use in EDDY in the preprocessing step):** *mouseID_sex*/*timepoint*/uFA_2Shapes_1A_3Rep_TR10/uFA_2Shapes_1A_3Rep_TR10_aveComb_preproc_mask *(in vivo)* *mouseID_sex*/*timepoint*/uFA_res130150500/uFA_res130150500_aveComb_preproc_mask *(ex vivo)*	.nii.gz
DiffusionDataPreproc	**Final brain mask:** *mouseID_sex*/*timepoint*/uFA_2Shapes_1A_3Rep_TR10/uFA_2Shapes_1A_3Rep_TR10_aveComb_preproc_mask_after *(in vivo)* *mouseID_sex*/*timepoint*/uFA_res130150500/uFA_res130150500_aveComb_preproc_mask_after *(ex vivo)*	.nii.gz
DiffusionDataPreproc	**Mean b0 volume extracted from dataset after preprocessing:** *mouseID_sex*/*timepoint*/uFA_2Shapes_1A_3Rep_TR10/uFA_2Shapes_1A_3Rep_TR10_aveComb_preproc_mean_b0 *(in vivo)*	.nii.gz

***Templates and Atlas for Registration***

**Table:** 

Folder	Filename	Filename Extensions
Registration	**Turone Atlas (as downloaded from** https://www.nitrc.org/projects/tmbta_2019/**):** atlas/TMBTA_Brain_Template **Turone Atlas Labels (as downloaded from** https://www.nitrc.org/projects/tmbta_2019/**):** atlas/TMBTA_Brain_Labels	.nii
Registration	**Downsampled Turone Atlas (to be used for registration):** atlas/TMBTA_Brain_Template_reorient_smoothed0_2_RS_Gaussian **Downsampled Turone Atlas Labels:**atlas/TMBTA_Brain_Labels_reorient_RS_Gaussian	.nii.gz
Registration	**Study-specific templates:** ANTStemplate_T2/T2_template (T2-weighted template)ANTStemplate_MT/MT_template (MTw template) ANTStemplate_FA/FA_template (FA template)	.nii.gz
Registration	**Registration transforms (Affine transform to register individual images to template space):** In each template folder: template_*contrast*_*mouseID*_*sex*_timepoint***GenericAffine where *‘contrast’* is ‘T2,’ ‘b2000_FA,’ or ‘MTon’ and *** are 3 numbers outputted by the ANTs template building command For example: template_T2_NR1_F_1week600GenericAffine	.mat
Registration	**Registration transforms (Symmetric diffeomorphic transform to register individual images to template space):** In each template folder: template_*contrast*_*mouseID*_*sex*_timepoint***Warp template_*contrast*_*mouseID*_*sex*_timepoint***InverseWarp	.nii.gz
Registration	**Registration transforms (between templates):** **FA template to T2 template:** FAtemplate_to_T2template/FA_T2_SynMI0_005_transform0GenericAffine.mat (affine transform) FAtemplate_to_T2template/FA_T2_SynMI0_005_transform1Warp.nii.gz (symmetric diffeomorphic transform) FAtemplate_to_T2template/FA_T2_SynMI0_005_transform1InverseWarp.nii.gz (inverse symmetric diffeomorphic transform) **MTw template to T2 template:** MTtemplate_to_T2template/ MT_T2_SynCI0.005_transform0GenericAffine.mat (affine transform) MTtemplate_to_T2template/ MT_T2_SynCI0.005__transform1Warp.nii.gz (symmetric diffeomorphic transform) MTtemplate_to_T2template/ MT_T2_SynCI0.005__transform1InverseWarp.nii.gz (inverse symmetric diffeomorphic transform)	
Registration	**Registration transforms (from T2 template to the downsampled atlas):** T2template_to_atlas/T2_atlas_SynMI0_00005_transform0GenericAffine.mat (affine transform) T2template_to_atlas/T2_atlas_SynMI0_00005_transform1Warp.nii.gz (symmetric diffeomorphic transform)	

### Imaging protocols

To optimize utility of the protocols, imaging protocols were exported from a Bruker ParaVision 6.0.1 system (OGSE and µA dMRI protocols), which was used for data collection, and are included. The files can be imported into the Bruker ParaVision system to run all protocols. ParaVision 6.0.1 compiled binaries for the custom diffusion MRI pulse sequences are available at doi.org/10.17605/OSF.IO/5EUSW, while the other scans used vendor-provided sequences. Imaging protocols and compiled binaries for a Bruker ParaVision 7.0.0 system are also included, for convenience. The diffusion MRI pulse sequence source code is available upon reasonable request.

## Technical Validation

**Fig. 6:**
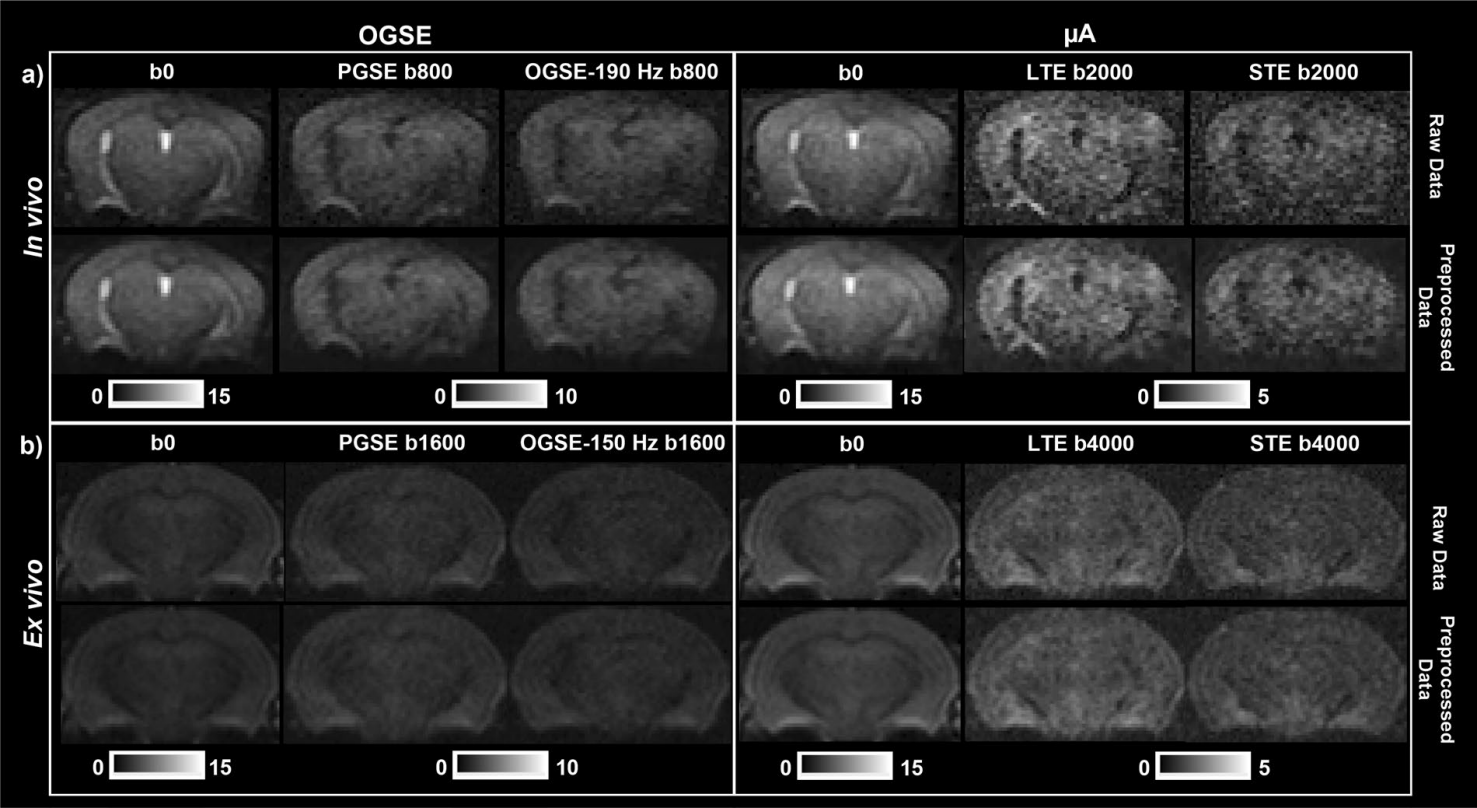
*In vivo* (**a**) and *ex vivo* (**b**) raw and preprocessed dMRI data. Raw data (after combining averages) is shown in the top row and preprocessed data is shown in the bottom row. Representative b = 0 s/mm^2^ images are shown for both the OGSE and µA protocols. From the OGSE protocol, representative diffusion weighted images from a single diffusion gradient direction are shown from PGSE and OGSE with the highest frequency used in this study (190 Hz (*in vivo*) and 150 Hz (*ex vivo*)), at b = 800 s/mm^2^ (*in vivo*) and b = 1600 s/mm^2^ (*ex vivo*). From the µA protocol, diffusion weighted images from a single diffusion gradient direction are shown from the LTE and STE sequences, at b = 2000 s/mm^2^ (*in vivo*) and b = 4000 s/mm^2^ (*ex vivo*). *Adapted from Rahman et al*.^2^.

As 3D printed custom designed parts and the surface/volume coil were fixed onto a support, which was placed into the scanner, this ensured consistent positioning of the mouse head in the scanner at each session and prevented motion artifacts. Raw and preprocessed dMRI data were visually inspected to ensure good preprocessing results, as shown previously^2^ and in Fig. 6a (*in vivo*) and 6b (*ex vivo*).

The only artifact observed in the *in vivo* data was a banding artifact in the rostral region of the brain in most of the B1 maps, which were acquired as part of the MT protocol. Thus, the MTsat maps included in the repository were generated without applying the B1 correction. Users have the option to turn the B1 correction on or off. If the B1 correction is on, the correction will be applied only to the slices which showed no banding artifact in the B1 map. Although the B1 maps have an artifact and the correction cannot be applied to all brain slices, inhomogeneities in the transmitted RF field are inherently compensated to some degree when calculating MTsat^6^. The B1 maps were acquired to correct for small residual higher-order dependencies of the MT saturation on the local RF transmit field to further improve spatial uniformity, as suggested by Weiskpof *et al*.^42^. Thus, the B1 correction is a finetuning for MTsat maps rather than a substantial part of the calculation, and the MTsat maps can still be analyzed without the correction.

For *ex vivo* data, as mouse IDs NR7_M and NR8_M were scanned with the skull removed, slight deformation of the tissue is observed at the superior edges of the brain. Mouse IDs NR1_F and NR2_F show banding artifacts in the caudal region of the brain in the B1 maps.

### Test-retest reproducibility

Test-retest analysis is an additional tool for technical validation. Test-retest comparisons have been performed using data from two timepoints: Day 3 and Week 1^2,3^. Bland-Altman plots and coefficients of variation (CVs) revealed that most of the μA dMRI metrics are reproducible in both ROI-based and voxelwise analysis, while the OGSE dMRI metrics are only reproducible in ROI-based analysis. MTR and MTsat show high reproducibility (CVs < 10%) in both voxelwise and ROI-based analyses. The previous test-retest analysis also shows that given feasible preclinical sample sizes (10–15), the MRI metrics may provide sensitivity to subtle microstructural changes (6–8%).

### Signal-to-noise ratio measurements

For dMRI data, SNR maps were calculated by dividing the powder-averaged magnitude signal (of the combined averages) by the noise. Noise was calculated from each of the real and imaginary components of the complex-valued data as the standard deviation of the background signal from a single average of a single direction divided by $\sqrt{\left({\rm{number}}\,{\rm{of}}\,{\rm{averages}}\right)\cdot \left({\rm{number}}\,{\rm{of}}\,{\rm{directions}}\right)}$, and averaged over the real and imaginary components. For MT data, SNR maps were calculated by dividing the magnitude signal by the standard deviation of background signal.

**Fig. 7:**
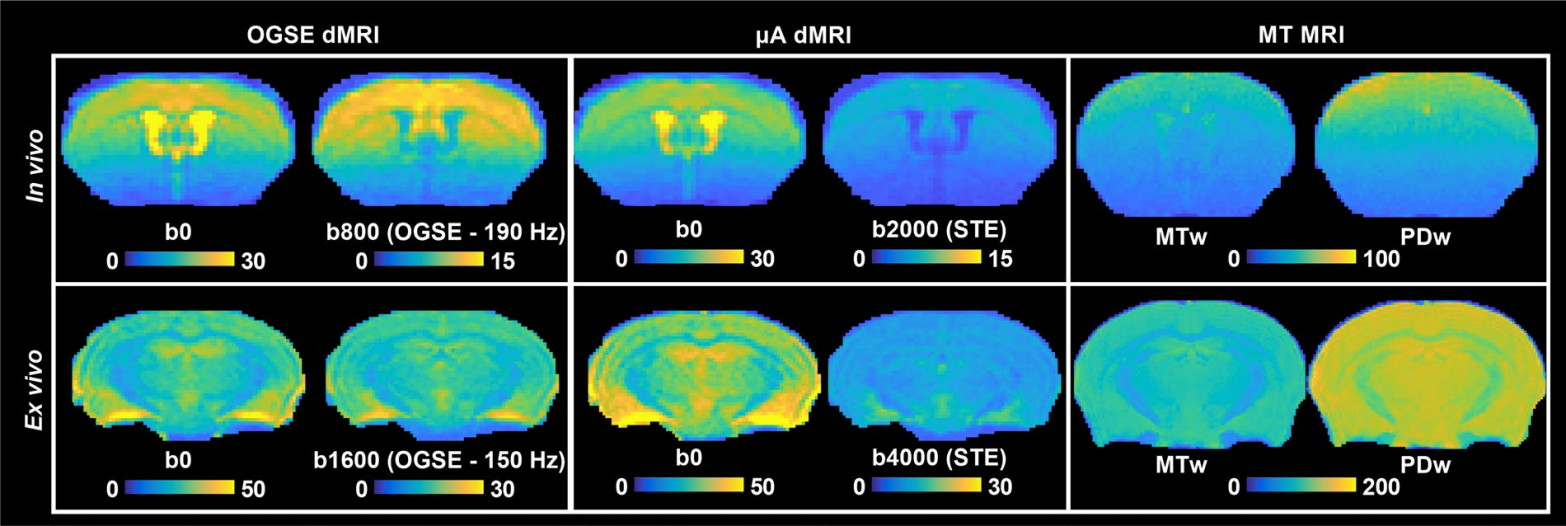
SNR maps of *in vivo* and *ex vivo* images. SNR maps for a single b = 0 s/mm^2^ image are shown for all dMRI protocols, and SNR maps for the powder average of the highest b-values are shown for all protocols (b = 800 s/mm^2^ for OGSE-190 Hz (*in vivo*), b = 2000 s/mm^2^ for μA-STE (*in vivo*), b = 1600 s/mm^2^ for OGSE-150 Hz (*ex vivo*), and b = 4000 s/mm^2^ for μA-STE (*ex vivo*)). SNR maps for MTw and PDw scans are shown for MT MRI. *Adapted from Rahman et al*.^2^.

To maximize SNR, a surface coil, built in-house, was used for *in vivo* imaging. As expected with a surface coil, a gradient of SNR change can be seen in the superior-inferior direction of the brain, compared to the commercially available MP30 volume coil, which was used for *ex vivo* imaging (Fig. 7). This gradient of SNR change does not seem to affect voxel-wise CV maps to the same extent, as shown in Rahman *et al*.^2^, which could be due to the denoising quality.

## Usage Notes

### Data analysis pipeline - snakemake

The data preprocessing and analysis pipeline was built using Snakemake^34^, a reproducible and adaptable Python-based workflow management system. Snakemake itself is easily deployable via the Conda package manager (https://conda.io). Instructions and further information can be found at https://snakemake.github.io.

The workflow, called the “Snakefile,” contains all data analysis steps such as DICOM to NIFTI conversion, preprocessing data, and scalar map generation. This involves FSL^31^, MRtrix3^38^, and ANTs^40^ commands, as well as MATLAB functions and bash scripts. Users can easily modify and add rules to the pipeline.

#### Example snakemake usage

Below are example Snakemake commands, which can be run from the command line, to process dicoms to preprocessed data and scalar maps. These instructions have also been included in the README of the code directory. The Snakemake rules used are listed directly below each command. The filepaths and filenames, which the user must change, are in italics, and any number of files can be converted at once. Importantly, most of the code assumes that the dicom or NIFTI filename matches the name of the folder that it is in. All brain masks (for each MRI contrast) have been provided in the repository and the user should copy the masks to their respective folders, as the code assumes that these masks exist. Alternatively, the user can edit the code to run without masks.

#### Anatomical data

To convert the anatomical dicoms (which include T2 and all MT related dicoms) to NIFTI format, the following Snakemake command can be used:

$ snakemake --cores 1 *filepath/*{*mouse#1_sex/timepoint*,*mouse#2_sex/timepoint*,*mouse#3_sex/timepoint*}/*dicom_foldername*/*dicom_filename*.json

[Rules: dcmTOnii_anat]

For example, a real use case of the above command, with the actual filepaths and filenames to acquire T2-weighted NIFTIs may be:

$ snakemake --cores 1 Data/{NR1_F/Day0,NR1_F/Day3,NR2_F/Day0}/T2_TurboRARE_AX150150500_A16/T2_TurboRARE_AX150150500_A16.json

Before acquiring MT metric maps, users must make a brain mask using the MT-weighted images (with software such as BrainSuite) and save the mask as “MTon_GRE_3D_150 × 400_12A_5uT_385FA_3500Hz_mask.nii.gz” in the folder “MTon_GRE_3D_150 × 400_12A_5uT_385FA_3500Hz.” As brain masks are also provided in the repository, users can also copy the mask, instead of creating a new one. To generate MT metric maps (MTR and MTsat), the following Snakemake command can be used:

$ snakemake --cores 1 *filepath/*{*mouse#1_sex /timepoint*,*mouse#2_sex /timepoint*,*mouse#3_sex /timepoint*}/ MTon_GRE_3D_150 × 400_12A_5uT_385FA_3500Hz/MTon_GRE_3D_150 × 400_12A_5uT_385FA_3500Hz_mtsat.nii.gz

[Rules: mtsat]

#### Diffusion MRI data

To convert a number of dicoms to combined averages (in NIFTI format, with partial Fourier reconstruction, correction for frequency and signal drift, and denoising) and generate the initial dMRI brain mask (needed for preprocessing), the following Snakemake command can be used:

$ snakemake --cores 1 *filepath/*{*mouse#1_sex /timepoint*,*mouse#2_sex /timepoint*,*mouse#3_sex /timepoint*}/*dMRI_filename*/*dMRI_filename*_aveComb_preproc_mask.nii.gz

[Rules: dcmTOnii_dMRI, combAve, get_preproc_mask]

The above command assumes that T2-weighted brain masks exist as “T2_TurboRARE_AX150150500_A16_mask.nii.gz” in the folder “T2_TurboRARE_AX150150500_A16,” as this mask is registered to dMRI space to create the initial dMRI brain mask. As the data acquired with reverse phase-encoding do not require an initial mask, since they are combined with the larger datasets (“uFA_2Shapes_1A_3Rep_TR10” and “OGSE_5Shapes_1A_5Rep_TR10”), the command to convert dicoms with reverse phase-encoding to combined averages is:

$ snakemake --cores 1 *filepath/*{*mouse#1_sex /timepoint*,*mouse#2_sex /timepoint*,*mouse#3_sex /timepoint*}/*dMRI_filename*/*dMRI_filename*_aveComb.nii.gz

[Rules: dcmTOnii_dMRI, combAve]

After combined averages and initial dMRI brain masks are generated, preprocessing can be run by this command:

$ snakemake --cores 1 DiffusionDataPreproc*/*{*mouse#1_sex /timepoint*,*mouse#2_sex /timepoint*,*mouse#3_sex /timepoint*}/*dMRI_filename*/*dMRI_filename*_aveComb_preproc.nii.gz

[Rules: dMRIpreproc]

Note that the code assumes that the original NIFTI files are located in the “Data” folder and that FSL is being run from a singularity container. The user can change the code in “dMRIpreproc.sh” located in the folder “code_scidata_paper/dMRIpreproc” to align with their FSL environment. The above command will work with or without reverse phase-encoded data.

After the dMRI preprocessing step, final dMRI brain masks can be created, or the user can use the masks provided in the repository (“*dMRI_filename*_aveComb_preproc_mask_after.nii.gz”). The code assumes that the masks are named as they are in the repository. Alternatively, the user can acquire dMRI scalar maps without using brain masks by editing the code in the Snakefile. To acquire dMRI scalar maps, the following command can be run:

$ snakemake --cores 1 *filepath/*{*mouse#1_sex /timepoint*,*mouse#2_sex /timepoint*,*mouse#3_sex /timepoint*}/*dMRI_filename*/*dMRI_filename*_aveComb_preproc_mean_b0_Wmask.nii.gz

[Rules: get_dwimetric_maps]

The above command will generate scalar maps as well as a non-diffusion weighted (b0) NIFTI, averaged over all non-diffusion weighted volumes. This mean b0 NIFTI may be used to facilitate registration.

### Image registration

The dMRI and MT data were not registered to a template or to the anatomical T2-weighted images to avoid errors from interpolation and registration inaccuracies, and as other researchers may prefer using their own registration pipelines. However, for flexible utility of the dataset, the anatomical T2-weighted images, study-specific templates, a downsampled atlas, and registration transforms have been included in the repository, so registration of the dMRI and MT data to anatomical space or an atlas is possible. Currently, the registration pipeline has been tested only with the *in vivo* dataset. Users may use the robust registration pipeline, based on ANTs commands, included in the Snakefile or tweak them accordingly. ANTs is an open source software package which comprises tools for image registration, template building and segmentation^40^. ANTs was chosen due to its flexibility and the robust performance of default ANTs registration parameters. Moreover, the nonlinear deformation algorithm used in ANTs was top ranked in a comparative study^43^.

**Fig. 8:**
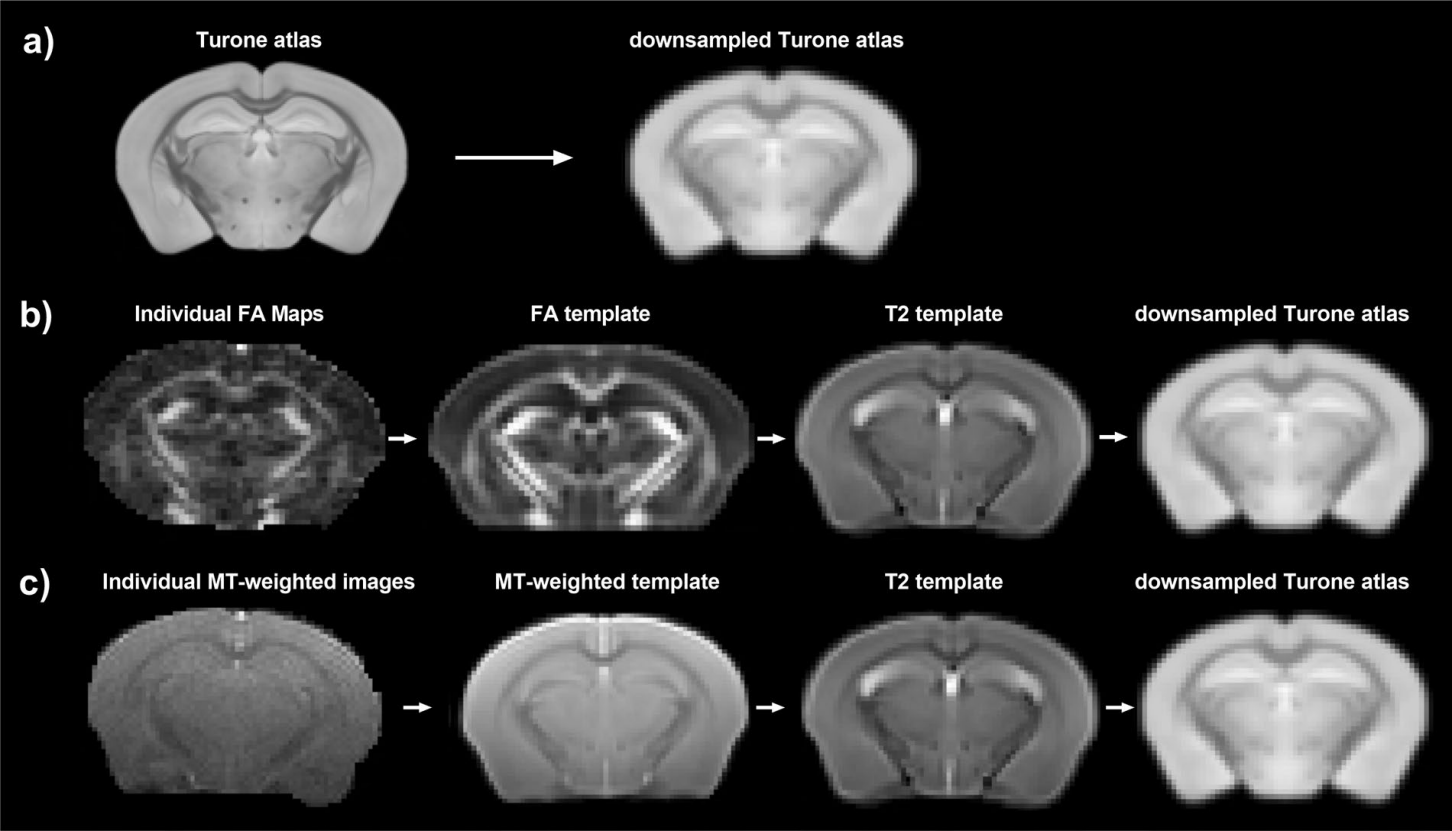
Schematic of registration steps. (**a**) The Turone atlas (60 µm isotropic resolution) was downsampled to the resolution of the T2-weighted images. (**b**) Registration steps to register individual FA maps to the downsampled atlas space. (**c**) Registration steps to register individual MT-weighted images to the downsampled atlas space. The registration transforms resulting from part (**b**,**c**) can be used to warp dMRI and MT metric maps to the downsampled atlas space.

The Turone atlas^44^, downsampled to the resolution of the *in vivo* T2-weighted images, was used for registration (Fig. 8a). Three study-specific templates, based on all images from all sessions, were created to facilitate the registration process. These templates included a T2 template, an FA template, and an MT-weighted template. Individual images can be registered to the downsampled atlas in three steps, as shown in Fig. 8b,c: (1) the FA maps and MT-weighted images are registered to the FA template and MT-weighted template, respectively; (2) the FA template and MT-weighted template are registered to the T2 template; (3) the T2 template is registered to the downsampled atlas. Each registration step involves an affine transformation, followed by a symmetric diffeomorphic transformation using ANTs’ Symmetric Normalization (SyN) algorithm. The registration transforms resulting from the previous three registration steps can be used to warp all dMRI metric maps and MT metric maps (MTR and MTsat) to the downsampled atlas space. Registration of all images to the atlas allows for voxel-wise analysis and atlas-based region-of-interest analysis. For region-of-interest analysis, atlas labels can be downloaded from https://www.nitrc.org/projects/tmbta_2019/^45^. Example ANTs commands, used for template-building and generating registration transforms, have been included in the text file ‘ANTs_Registration_Commands.txt.’ All other code to warp metric maps to the downsampled atlas space have been included in the Snakefile.

## Supplementary information

Supplementary Table 1

## Data Availability

As mentioned previously, all code required to process dicoms to the final scalar maps is available: 10.20383/103.0594^45^. The code is also available publicly through GitLab: https://gitlab.com/cfmm/pipelines/mouse_dmri_MT_dicomTOscalarMaps. This includes a Snakemake pipeline, which includes FSL, MRtrix3, and ANTs commands, and MATLAB functions. The custom dMRI pulse sequences used in this work are available as binary methods: https://osf.io/5eusw/, and the source code is available upon reasonable request^46^.

## References

[1] Hammelrath L (2016). Morphological maturation of the mouse brain: An *in vivo* MRI and histology investigation. Neuroimage.

[2] Rahman N (2021). Test-retest reproducibility of *in vivo* oscillating gradient and microscopic anisotropy diffusion MRI in mice at 9.4 Tesla. PLoS One.

[3] Rahman, N., Ramnarine, J., Xu, K., Brown, A. & Baron, C. Test-retest reproducibility of *in vivo* magnetization transfer ratio and saturation index in mice at 9.4 Tesla. *J Magn Reson Imaging* 1–11, 10.1002/jmri.28106 (2022). 10.1002/jmri.28106 35156740

[4] Schmierer K, Scaravilli F, Altmann DR, Barker GJ, Miller DH (2004). Magnetization transfer ratio and myelin in postmortem multiple sclerosis brain. Ann. Neurol..

[5] Gass A (1994). Correlation of magnetization transfer ration with clinical disability in multiple sclerosis. Ann. Neurol..

[6] Helms G, Dathe H, Kallenberg K, Dechent P (2008). High-resolution maps of magnetization transfer with inherent correction for RF inhomogeneity and T1 relaxation obtained from 3D FLASH MRI. Magn. Reson. Med..

[7] Lema A (2017). A Comparison of Magnetization Transfer Methods to Assess Brain and Cervical Cord Microstructure in Multiple Sclerosis. J. Neuroimaging.

[8] Jones DK, Knösche TR, Turner R (2013). White matter integrity, fiber count, and other fallacies: The do’s and don’ts of diffusion MRI. Neuroimage.

[9] Baron CA, Beaulieu C (2014). Oscillating gradient spin-echo (OGSE) diffusion tensor imaging of the human brain. Magn. Reson. Med..

[10] Xu J (2021). Probing neural tissues at small scales: Recent progress of oscillating gradient spin echo (OGSE) neuroimaging in humans. J. Neurosci. Methods.

[11] Lasič S, Szczepankiewicz F, Eriksson S, Nilsson M, Topgaard D (2014). Microanisotropy imaging: Quantification of microscopic diffusion anisotropy and orientational order parameter by diffusion MRI with magic-angle spinning of the q-vector. Front. Phys..

[12] Ianuş A (2018). Accurate estimation of microscopic diffusion anisotropy and its time dependence in the mouse brain. Neuroimage.

[13] Shemesh N (2016). Conventions and nomenclature for double diffusion encoding NMR and MRI. Magn. Reson. Med..

[14] Arezza NJJ, Tse DHY, Baron CA (2021). Rapid microscopic fractional anisotropy imaging via an optimized linear regression formulation. Magn. Reson. Imaging.

[15] Novikov DS, Jensen JH, Helpern JA, Fieremans E (2014). Revealing mesoscopic structural universality with diffusion. Proc. Natl. Acad. Sci. USA.

[16] Does MD, Parsons EC, Gore JC (2003). Oscillating gradient measurements of water diffusion in normal and globally ischemic rat brain. Magn. Reson. Med..

[17] Arbabi A, Kai J, Khan AR, Baron CA (2020). Diffusion dispersion imaging: Mapping oscillating gradient spin-echo frequency dependence in the human brain. Magn. Reson. Med..

[18] Mitra PP (1995). Multiple wave-vector extensions of the NMR pulsed-field-gradient spin-echo diffusion measurement. Am. Phys. Soc..

[19] Nilsson M (2020). Tensor-valued diffusion MRI in under 3 minutes: an initial survey of microscopic anisotropy and tissue heterogeneity in intracranial tumors. Magn. Reson. Med..

[20] Szczepankiewicz F (2015). Quantification of microscopic diffusion anisotropy disentangles effects of orientation dispersion from microstructure: Applications in healthy volunteers and in brain tumors. Neuroimage.

[21] Ianuş A, Drobnjak I, Alexander DC (2016). Model-based estimation of microscopic anisotropy using diffusion MRI: A simulation study. NMR Biomed..

[22] Özarslan E (2009). Compartment shape anisotropy (CSA) revealed by double pulsed field gradient MR. J. Magn. Reson..

[23] Cheng Y, Cory DG (1999). Multiple scattering by NMR. J. Am. Chem. Soc..

[24] Jespersen SN, Lundell H, Sønderby CK, Dyrby TB (2013). Orientationally invariant metrics of apparent compartment eccentricity from double pulsed field gradient diffusion experiments. NMR Biomed..

[25] Shemesh N, Cohen Y (2011). Microscopic and compartment shape anisotropies in gray and white matter revealed by angular bipolar double-PFG MR. Magn. Reson. Med..

[26] Tu TW (2016). Radiological-pathological correlation of diffusion tensor and magnetization transfer imaging in a closed head traumatic brain injury model. Ann. Neurol..

[27] Tu TW (2017). Abnormal injury response in spontaneous mild ventriculomegaly wistar rat brains: A pathological correlation study of diffusion tensor and magnetization transfer imaging in mild traumatic brain injury. J. Neurotrauma.

[28] Wu TL (2020). Longitudinal assessment of recovery after spinal cord injury with behavioral measures and diffusion, quantitative magnetization transfer and functional magnetic resonance imaging. NMR Biomed..

[29] Andersson JLR, Skare S, Ashburner J (2003). How to correct susceptibility distortions in spin-echo echo-planar images: Application to diffusion tensor imaging. Neuroimage.

[30] Andersson JLR, Sotiropoulos SN (2016). An integrated approach to correction for off-resonance effects and subject movement in diffusion MR imaging. Neuroimage.

[31] Smith SM (2004). Advances in functional and structural MR image analysis and implementation as FSL. Neuroimage.

[32] Borsos, K. B., Tse, D. H. Y., Dubovan, P. I. & Baron, C. A. Frequency tuned bipolar oscillating gradients for mapping diffusion kurtosis dispersion in the human brain. 1–11, 10.1002/mrm.29473 (2022). 10.1002/mrm.29473 36198030

[33] Yarnykh VL (2007). Actual flip-angle imaging in the pulsed steady state: A method for rapid three-dimensional mapping of the transmitted radiofrequency field. Magn. Reson. Med..

[34] Köster, J. *et al*. Sustainable data analysis with Snakemake. *F1000Research* **10** (2021). 10.12688/f1000research.29032.1 PMC8114187 34035898

[35] Haacke EM, Lindskog ED, Lin W (1991). A fast, iterative, partial-fourier technique capable of local phase recovery. J. Magn. Reson..

[36] Vos SB (2017). The importance of correcting for signal drift in diffusion MRI. Magn. Reson. Med..

[37] Veraart J (2016). Denoising of diffusion MRI using random matrix theory. Neuroimage.

[38] Tournier JD (2019). MRtrix3: A fast, flexible and open software framework for medical image processing and visualisation. Neuroimage.

[39] Shattuck DW, Leahy RM (2000). Brainsuite: An automated cortical surface identification tool. Lect. Notes Comput. Sci. (including Subser. Lect. Notes Artif. Intell. Lect. Notes Bioinformatics).

[40] Avants BB (2011). A reproducible evaluation of ANTs similarity metric performance in brain image registration. Neuroimage.

[41] Hagiwara A (2018). Myelin Measurement: Comparison between Simultaneous Tissue Relaxometry, Magnetization Transfer Saturation Index, and T1w/T2w Ratio Methods. Sci. Rep..

[42] Weiskopf N (2013). Quantitative multi-parameter mapping of R1, PD*, MT, and R2* at 3T: A multi-center validation. Front. Neurosci..

[43] Rahman, N., Xu, K., Budde, M., Brown, A. & Baron, C. A longitudinal microstructural MRI dataset in healthy C57Bl/6 mice at 9.4 Tesla. *Federated Research Data Repository*10.20383/103.0594 (2022). 10.1038/s41597-023-01942-5 PMC9929084 36788251

[44] Klein A (2009). Evaluation of 14 nonlinear deformation algorithms applied to human brain MRI registration. Neuroimage.

[45] Barriere, D. A. Turone Mouse Brain Template and Atlas. *NeuroImaging Tools and Research Collaboratory* https://www.nitrc.org/projects/tmbta_2019/ (2019).

[46] Baron C (2021). Baron Lab Pulse Sequences..

