# Supplementary material for: A longitudinal microstructural MRI dataset in healthy C57Bl/6 mice at 9.4 Tesla
Source: Sci Data. 2023 Feb 14;10:94. doi: 10.1038/s41597-023-01942-5 (PMC9929084; doi:10.1038/s41597-023-01942-5)
Supplement: Supplementary file 1 — Supplementary Table 1 [file 41597_2023_1942_MOESM1_ESM.docx]

**Supplementary Tables**

| Imaging Protocol | MRI Metrics (Scalar Maps) | Equation | Interpretation |
| --- | --- | --- | --- |
| MT | Magnetization transfer ratio ($\mathrm{MTR}$) | $MTR= \frac{PDw-MTw}{\mathrm{PDw}}$ | MTR has been shown to correlate well with histological myelin content.^1,2^ However, MTR is also sensitive to the choice of sequence parameters, flip angle inhomogeneities, and longitudinal relaxation time (T1).^3^ |
|  | Magnetization transfer saturation index ($\mathrm{MTsat}$) | The apparent MTsat is calculated as:  $\mathrm{MTsat}_{\mathrm{app}}=\left( \frac{A_{\mathrm{app}}\alpha_{\mathrm{MT}}}{S_{\mathrm{MT}}}-1 \right)R_{1app}\mathrm{TR}_{\mathrm{MT}}- \alpha_{\mathrm{MT}}^{2}/2$  $A_{\mathrm{app}}$: apparent signal amplitude; $\alpha_{\mathrm{MT}}$: excitation flip angle of the MTw image; $S_{\mathrm{MT}}$: signal intensity of MTw image; $R_{1app}$: apparent longitudinal relaxation rate; $\mathrm{TR}_{\mathrm{MT}}$: TR of MTw image  $\mathrm{MTsat}$ was calculated from $\mathrm{MTsat}_{\mathrm{app}}$, after correcting for small residual higher order dependencies of the MT saturation on the local RF transmit field to further improve spatial uniformity:  $MTsat= \frac{\mathrm{MTsat}_{\mathrm{app}}\cdot(1 - 0.4)}{1 - 0.4\cdot\mathrm{RF}_{\mathrm{local}}}$  $\mathrm{RF}_{\mathrm{local}}$: relative flip angle α compared to the nominal flip angle  For more details on the calculations of $A_{\mathrm{app}}$ and $R_{1app}$, see Helms et al.^3^ | MTsat reduces T1 dependence and improves specificity to myelin, compared to MTR, while maintaining a feasible scan time.^3^ |
| OGSE dMRI | DTI Metrics at each frequency:   - Axial Diffusivity ($\mathrm{AD}$) - Radial Diffusivity ($\mathrm{RD}$) - Mean Diffusivity ($\mathrm{AD}$) - Fractional Anisotropy ($\mathrm{FA}$) | DTI assumes Gaussian diffusion and models diffusion as an ellipsoid with three eigenvectors and corresponding eigenvalues ($\lambda_{1}$, $\lambda_{2}$, $\lambda_{3}$).  $AD=\lambda_{1}$  $RD=\frac{\lambda_{2}+\lambda_{3}}{2}$  $MD=\frac{{\lambda_{1}+\lambda}_{2}+\lambda_{3}}{3}$  $FA=\sqrt{\frac{3}{2}}\frac{\sqrt{{(\lambda_{1}-MD)}^{2}+{(\lambda_{2}-MD)}^{2}+{(\lambda_{3}-MD)}^{2}}}{\sqrt{{\lambda_{1}}^{2}{{+\lambda}_{2}}^{2}+{\lambda_{3}}^{2}}}$ | DTI metrics reflect white matter tissue properties such as myelination or fibre density. However, the effects of orientation dispersion dominate such contrast and more sophisticated models are necessary to separate the effects.^4^ |
|  | Mean diffusivity difference ($\Delta MD$) | $\Delta MD=\mathrm{MD}_{f}-\mathrm{MD}_{0}$  $\mathrm{MD}_{f}$: OGSE $\mathrm{MD}$ at a frequency $f$  $\mathrm{MD}_{0}$: $\mathrm{MD}$ at $f$ = 0  $f$: OGSE frequency | Here, $\Delta MD$ maps provided are $\Delta MD$ between OGSE-190 Hz and PGSE-0 Hz. $\Delta MD$ has shown increased sensitivity, compared to MD alone, in the assessment of hypoxia-ischemia in rodents,^5^ and in various pathologies in humans.^6–8^ Notably, $\Delta MD$ has helped to identify neurite beading as a mechanism for dMRI contrast after ischemic stroke.^9,10^ |
|  | Diffusion dispersion rate ($\Lambda$) | $\mathrm{MD}_{f}=\mathrm{MD}_{0}+\Lambda f^{0.5}$ | By acquiring diffusion data at multiple frequencies, the power law relationship between MD and frequency can be explored via the “diffusion dispersion rate”, $\Lambda$.^11,12^ |
| µA dMRI | DTI Metrics (acquired with b1000 LTE volumes):  AD, RD, MD, FA |  |  |
|  | Microscopic anisotropy ($\mu A$) | $\mu A= \sqrt{\frac{ln(\frac{S_{\mathrm{LTE}}}{S_{\mathrm{STE}}})}{b^{2}}}$  $S_{\mathrm{LTE}}$: powder-averaged LTE signal  $S_{\mathrm{STE}}$: powder-averaged STE signal | In contrast to the widely used FA metric, which confounds true microstructural changes with fiber orientation dispersion,^1^ the microscopic anisotropy ($\mu A$) metric quantifies water diffusion anisotropy independent of orientation dispersion.^13,14^ $\mu A$ is defined here based on the difference in signal between LTE and STE dMRI acquisitions. Preliminary studies in humans have found that $\mu A$ provides better sensitivity than the conventional $FA$ in distinguishing between different types of brain tumours,^15^ and the assessment of multiple sclerosis lesions.^16,17^ |
|  | Microscopic fractional anisotropy ($\mu FA$) | $\mu FA= \sqrt{\frac{3}{2}\frac{{\mu A}^{2}}{{\mu A}^{2}+0.2\mathrm{MD}^{2}}}$ | $\mu FA$ is the normalized counterpart of $\mu A$ and can be expressed in terms of $\mu A$. |
|  | Linear Diffusion Kurtosis ($K_{\mathrm{LTE}}$) | $K_{\mathrm{LTE}}$ was calculated by fitting the LTE signal to the diffusion kurtosis model using a joint non-negative least squares method, assuming $\mathrm{MD}$ is the same between LTE and STE acquisitions:  $\ln\left( \frac{S_{\mathrm{LTE}}}{S_{o}} \right)=-bMD+\frac{1}{6}b^{2}MD^{2}K_{\mathrm{LTE}}$  $S_{o}$: mean signal with no diffusion encoding | $K_{\mathrm{LTE}}$ arises from the LTE acquisitions, which depends on the variance of both isotropic and anisotropic diffusivity. |
|  | Isotropic diffusion kurtosis ($K_{\mathrm{STE}}$) | $K_{\mathrm{STE}}$ was calculated as described above:  $\ln\left( \frac{S_{\mathrm{STE}}}{S_{o}} \right)=-bMD+\frac{1}{6}b^{2}MD^{2}K_{\mathrm{STE}}$ | $K_{\mathrm{STE}}$ arises from the STE acquisitions, which depends only on the variance of isotropic diffusivity. $K_{\mathrm{STE}}$ is a measure of the variance in the magnitude of diffusion tensors or the mean diffusivity, which can be related to cell size heterogeneity.^15^ Recently, He et al. have shown that $K_{\mathrm{STE}}$ may be particularly sensitive to deep gray matter lesions.^18^ |

**Supplementary Table 1.** Brief description of scalar maps provided in the repository.

**References**

1. Schmierer, K., Scaravilli, F., Altmann, D. R., Barker, G. J. & Miller, D. H. Magnetization transfer ratio and myelin in postmortem multiple sclerosis brain. *Ann. Neurol.* **56**, 407–415 (2004).

2. Mottershead, J. P., Schmierer, K., Clemence, M., *et al.* High field MRI correlates of myelin content and axonal density in multiple sclerosis: A post-mortem study of the spinal cord. *J. Neurol.* **250**, 1293–1301 (2003).

3. Helms, G., Dathe, H., Kallenberg, K. & Dechent, P. High-resolution maps of magnetization transfer with inherent correction for RF inhomogeneity and T1 relaxation obtained from 3D FLASH MRI. *Magn. Reson. Med.* **60**, 1396–1407 (2008).

4. Jones, D. K., Knösche, T. R. & Turner, R. White matter integrity, fiber count, and other fallacies: The do’s and don’ts of diffusion MRI. *Neuroimage* **73**, 239–254 (2013).

5. Wu, D., Martin, L. J., Northington, F. J. & Zhang, J. Oscillating gradient diffusion MRI reveals unique microstructural information in normal and hypoxia-ischemia injured mouse brains. *Magn. Reson. Med.* **72**, 1366–1374 (2014).

6. Maekawa, T., Hori, M., Murata, K., *et al.* Differentiation of high-grade and low-grade intra-axial brain tumors by time-dependent diffusion MRI. *Magn. Reson. Imaging* **72**, 34–41 (2020).

7. Mazzoli, V., Moulin, K., Kogan, F., Hargreaves, B. A. & Gold, G. E. Diffusion Tensor Imaging of Skeletal Muscle Contraction Using Oscillating Gradient Spin Echo. *Front. Neurol.* **12**, 1–10 (2021).

8. Gao, F., Shen, X., Zhang, H., *et al.* Feasibility of oscillating and pulsed gradient diffusion MRI to assess neonatal hypoxia-ischemia on clinical systems. *J. Cereb. Blood Flow Metab.* (2020) doi:10.1177/0271678X20944353.

9. Budde, M. D. & Frank, J. A. Neurite beading is sufficient to decrease the apparent diffusion coefficient after ischemic stroke. *Proc. Natl. Acad. Sci. U. S. A.* **107**, 14472–14477 (2010).

10. Baron, C. A., Kate, M., Gioia, L., *et al.* Reduction of Diffusion-Weighted Imaging Contrast of Acute Ischemic Stroke at Short Diffusion Times. *Stroke.* **46**, 2136–2141 (2015).

11. Burcaw, L. M., Fieremans, E. & Novikov, D. S. Mesoscopic structure of neuronal tracts from time-dependent diffusion. *Neuroimage* **114**, 18–37 (2015).

12. Novikov, D. S., Jensen, J. H., Helpern, J. A. & Fieremans, E. Revealing mesoscopic structural universality with diffusion. *Proc. Natl. Acad. Sci. U. S. A.* **111**, 5088–5093 (2014).

13. Lasič, S., Szczepankiewicz, F., Eriksson, S., Nilsson, M. & Topgaard, D. Microanisotropy imaging: Quantification of microscopic diffusion anisotropy and orientational order parameter by diffusion MRI with magic-angle spinning of the q-vector. *Front. Phys.* **2**, 1–14 (2014).

14. Shemesh, N., Jespersen, S. N., Alexander, D. C., *et al.* Conventions and nomenclature for double diffusion encoding NMR and MRI. *Magn. Reson. Med.* **75**, 82–87 (2016).

15. Szczepankiewicz, F., Lasič, S., van Westen, D., *et al.* Quantification of microscopic diffusion anisotropy disentangles effects of orientation dispersion from microstructure: Applications in healthy volunteers and in brain tumors. *Neuroimage* **104**, 241–252 (2015).

16. Yang, G., Tian, Q., Leuze, C., Wintermark, M. & McNab, J. Double Diffusion Encoding MRI for the Clinic. *Magn. Reson. Med.* **80**, 507–520 (2018).

17. Andersen, K. W., Lasič, S., Lundell, H., *et al.* Disentangling white-matter damage from physiological fibre orientation dispersion in multiple sclerosis. *Brain Commun.* **2**, (2020).

18. He, Y., Aznar, S., Siebner, H. R. & Dyrby, T. B. In vivo tensor-valued diffusion MRI of focal demyelination in white and deep grey matter of rodents. *NeuroImage Clin.* **30**, 102675 (2021).
